# 4-Chloro-*N*-(2,3-dichloro­phen­yl)benzene­sulfonamide

**DOI:** 10.1107/S1600536810053638

**Published:** 2010-12-24

**Authors:** K. Shakuntala, Sabine Foro, B. Thimme Gowda

**Affiliations:** a Department of Chemistry, Mangalore University, Mangalagangotri 574 199, Mangalore, India; b Institute of Materials Science, Darmstadt University of Technology, Petersenstrasse 23, D-64287 Darmstadt, Germany

## Abstract

In the title compound, C_12_H_8_Cl_3_NO_2_S, the two aromatic rings are tilted relative to each other by 56.5 (1)°. The crystal structure features centrosymmetric dimers in which mol­ecules are linked by N—H⋯O hydrogen bonds.

## Related literature

For our study of the effect of substituents on the structures of *N*-(ar­yl)aryl­sulfonamides, see: Gowda *et al.* (2010 ▶); Nirmala *et al.* (2010 ▶); Shakuntala *et al.* (2010 ▶). For related structures, see: Gelbrich *et al.* (2007 ▶); Perlovich *et al.* (2006 ▶).
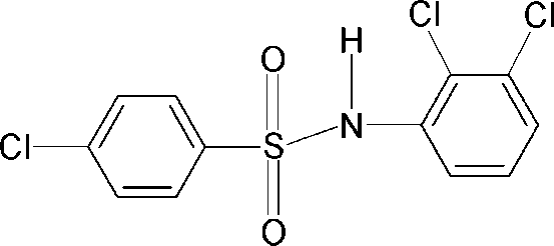


## Experimental

### 

#### Crystal data

C_12_H_8_Cl_3_NO_2_S
*M* *_r_* = 336.60
Monoclinic, 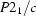

*a* = 7.224 (1) Å
*b* = 14.975 (2) Å
*c* = 13.170 (2) Å
β = 97.16 (1)°
*V* = 1413.6 (3) Å^3^
*Z* = 4
Cu *K*α radiation
μ = 7.23 mm^−1^
*T* = 299 K
0.38 × 0.30 × 0.20 mm

#### Data collection

Enraf–Nonius CAD-4 diffractometer
Absorption correction: ψ scan (North *et al.*, 1968 ▶) *T*_min_ = 0.170, *T*_max_ = 0.326
2767 measured reflections
2516 independent reflections
2277 reflections with *I* > 2σ(*I*)
*R*_int_ = 0.062
3 standard reflections every 120 min  intensity decay: 0.5%

#### Refinement

*R*[*F* ^2^ > 2σ(*F* ^2^)] = 0.043
*wR*(*F* ^2^) = 0.137
*S* = 1.15
2516 reflections
176 parameters
1 restraint
H atoms treated by a mixture of independent and constrained refinement
Δρ_max_ = 0.46 e Å^−3^
Δρ_min_ = −0.51 e Å^−3^

### 

Data collection: *CAD-4-PC* (Enraf–Nonius, 1996 ▶); cell refinement: *CAD-4-PC*; data reduction: *REDU4* (Stoe & Cie, 1987 ▶); program(s) used to solve structure: *SHELXS97* (Sheldrick, 2008 ▶); program(s) used to refine structure: *SHELXL97* (Sheldrick, 2008 ▶); molecular graphics: *PLATON* (Spek, 2009 ▶); software used to prepare material for publication: *SHELXL97*.

## Supplementary Material

Crystal structure: contains datablocks I, global. DOI: 10.1107/S1600536810053638/bt5445sup1.cif

Structure factors: contains datablocks I. DOI: 10.1107/S1600536810053638/bt5445Isup2.hkl

Additional supplementary materials:  crystallographic information; 3D view; checkCIF report

## Figures and Tables

**Table 1:** Hydrogen-bond geometry (Å, °)

*D*—H⋯*A*	*D*—H	H⋯*A*	*D*⋯*A*	*D*—H⋯*A*
N1—H1*N*⋯O1^i^	0.86 (2)	2.11 (2)	2.944 (3)	163 (3)

Symmetry code: (i) 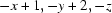.

**Fig. 1.**
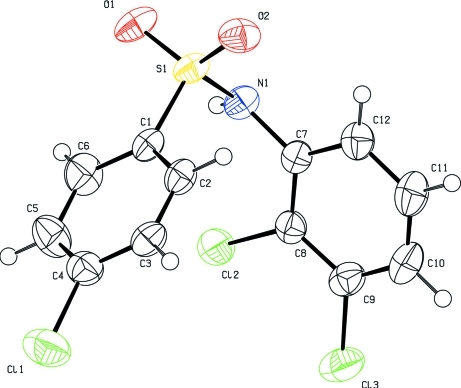
Molecular structure of the title compound, showing the atom labelling scheme and displacement ellipsoids are drawn at the 50% probability level.

**Fig. 2.**
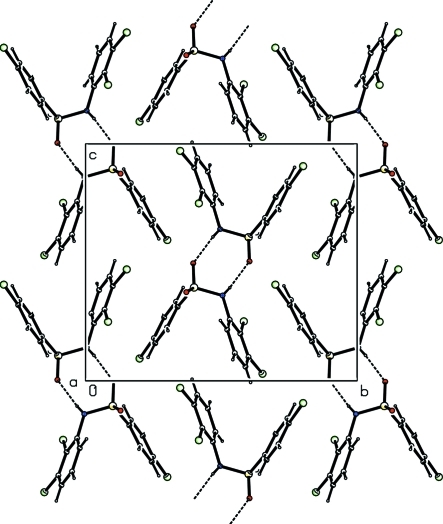
Molecular packing of the title compound with hydrogen bonding shown as dashed lines.

## supplementary crystallographic information

### Comment

As part of a study of the substituent effects on the crystal structures of *N*-(aryl)-arylsulfonamides (Gowda *et al.*, 2010; Nirmala *et al.*, 2010; Shakuntala *et al.*, 2010), in the present work, the structure of 4-chloro-*N*-(2,3-dichlorophenyl)-benzenesulfonamide (I) has been determined. The conformation of the N—C bond in the C—SO_2_—NH—C segment of the structure has *gauche* torsions with respect to the S═ O bonds (Fig. 1). The molecule is bent at the S atom with the C—SO_2_—NH—C torsion angle of -56.7 (2)°, compared to the values of 65.4 (2) and -61.7 (2) in the two molecules of *N*-(2,3-dichlorophenyl)- 4-methylbenzenesulfonamide (II) (Shakuntala *et al.*, 2010). The conformations of the N—H bond and the *ortho*-chloro group in the anilino benzene ring are *syn* to each other.

The sulfonyl and the anilino benzene rings in (I) are tilted relative to each other by 56.5 (1)°, compared to the values of 76.0 (1)° (molecule 1) and 79.9 (1)° (molecule 2) in (II).

The other bond parameters in (I) are similar to those observed in (II), *N*-(2,3-dichlorophenyl)-2,4-dimethylbenzenesulfonamide (Nirmala *et al.*, 2010), *N*-(3,4-dimethylphenyl)-4-chlorobenzene- sulfonamide (Gowda *et al.*, 2010) and other aryl sulfonamides, (Perlovich *et al.*, 2006; Gelbrich *et al.*, 2007).

The structure shows  N—H···O intermolecular H-bonding (Table 1). The crystal packing is shown in Fig. 2.

### Experimental

The solution of chlorobenzene (10 ml) in chloroform (40 ml) was treated dropwise with chlorosulfonic acid (25 ml) at 0 ° C. After the initial evolution of hydrogen chloride subsided, the reaction mixture was brought to room temperature and poured into crushed ice in a beaker. The chloroform layer was separated, washed with cold water and allowed to evaporate slowly. The residual 4-chlorobenzenesulfonylchloride was treated with 2,3-dichloroaniline in the stoichiometric ratio and boiled for ten minutes. The reaction mixture was then cooled to room temperature and added to ice cold water (100 ml). The resultant 4-chloro-*N*-(2,3-dichlorophenyl)- benzenesulfonamide was filtered under suction and washed thoroughly with cold water. It was then recrystallized to constant melting point from dilute ethanol. The purity of the compound was checked and characterized by recording its infrared and NMR spectra.

Prism like colorless single crystals used in X-ray diffraction studies were grown in ethanolic solution by slow evaporation at room temperature.

### Refinement

The H atom of the NH group was located in a diffrerence and its coordinates were refined with the N—H distance restrained to 0.86 (2) Å. The other H atoms were positioned with idealized geometry using a riding model [C—H = 0.93 Å]. All H atoms were refined with isotropic displacement parameters set to 1.2 times of the *U*_eq_ of the parent atom.

### Crystal data

**Table:**

C_12_H_8_Cl_3_NO_2_S	*F*(000) = 680
*M**_r_* = 336.60	*D*_x_ = 1.582 Mg m^−^^3^
Monoclinic, *P*2_1_/*c*	Cu *K*α radiation, λ = 1.54180 Å
Hall symbol: -P 2ybc	Cell parameters from 25 reflections
*a* = 7.224 (1) Å	θ = 6.8–22.5°
*b* = 14.975 (2) Å	µ = 7.23 mm^−^^1^
*c* = 13.170 (2) Å	*T* = 299 K
β = 97.16 (1)°	Prism, colourless
*V* = 1413.6 (3) Å^3^	0.38 × 0.30 × 0.20 mm
*Z* = 4	

### Data collection

**Table:**

Enraf–Nonius CAD-4 diffractometer	2277 reflections with *I* > 2σ(*I*)
Radiation source: fine-focus sealed tube	*R*_int_ = 0.062
graphite	θ_max_ = 66.9°, θ_min_ = 4.5°
ω/2θ scans	*h* = −8→8
Absorption correction: ψ scan (North *et al.*, 1968)	*k* = −17→0
*T*_min_ = 0.170, *T*_max_ = 0.326	*l* = −15→1
2767 measured reflections	3 standard reflections every 120 min
2516 independent reflections	intensity decay: 0.5%

### Refinement

**Table:**

Refinement on *F*^2^	Secondary atom site location: difference Fourier map
Least-squares matrix: full	Hydrogen site location: inferred from neighbouring sites
*R*[*F*^2^ > 2σ(*F*^2^)] = 0.043	H atoms treated by a mixture of independent and constrained refinement
*wR*(*F*^2^) = 0.137	*w* = 1/[σ^2^(*F*_o_^2^) + (0.0763*P*)^2^ + 1.0223*P*] where *P* = (*F*_o_^2^ + 2*F*_c_^2^)/3
*S* = 1.15	(Δ/σ)_max_ = 0.001
2516 reflections	Δρ_max_ = 0.46 e Å^−^^3^
176 parameters	Δρ_min_ = −0.51 e Å^−^^3^
1 restraint	Extinction correction: *SHELXL97* (Sheldrick, 2008), Fc^*^=kFc[1+0.001xFc^2^λ^3^/sin(2θ)]^-1/4^
Primary atom site location: structure-invariant direct methods	Extinction coefficient: 0.0074 (7)

### Special details

**Table:**

Geometry. All e.s.d.'s (except the e.s.d. in the dihedral angle between two l.s. planes) are estimated using the full covariance matrix. The cell e.s.d.'s are taken into account individually in the estimation of e.s.d.'s in distances, angles and torsion angles; correlations between e.s.d.'s in cell parameters are only used when they are defined by crystal symmetry. An approximate (isotropic) treatment of cell e.s.d.'s is used for estimating e.s.d.'s involving l.s. planes.
Refinement. Refinement of *F*^2^ against ALL reflections. The weighted *R*-factor *wR* and goodness of fit *S* are based on *F*^2^, conventional *R*-factors *R* are based on *F*, with *F* set to zero for negative *F*^2^. The threshold expression of *F*^2^ > σ(*F*^2^) is used only for calculating *R*-factors(gt) *etc*. and is not relevant to the choice of reflections for refinement. *R*-factors based on *F*^2^ are statistically about twice as large as those based on *F*, and *R*- factors based on ALL data will be even larger.

### Fractional atomic coordinates and isotropic or equivalent isotropic displacement parameters (Å^2^)

**Table:**

	*x*	*y*	*z*	*U*_iso_*/*U*_eq_	
C1	0.5255 (4)	0.83900 (18)	0.1877 (2)	0.0332 (6)	
C2	0.4733 (4)	0.80016 (19)	0.2749 (2)	0.0372 (6)	
H2	0.3502	0.8034	0.2886	0.045*	
C3	0.6057 (4)	0.7565 (2)	0.3415 (2)	0.0401 (7)	
H3	0.5726	0.7303	0.4008	0.048*	
C4	0.7869 (4)	0.7519 (2)	0.3199 (2)	0.0429 (7)	
C5	0.8394 (5)	0.7896 (3)	0.2323 (3)	0.0551 (9)	
H5	0.9622	0.7853	0.2183	0.066*	
C6	0.7079 (5)	0.8336 (2)	0.1657 (3)	0.0504 (8)	
H6	0.7412	0.8594	0.1064	0.060*	
C7	0.3738 (4)	1.03085 (17)	0.2425 (2)	0.0326 (6)	
C8	0.5279 (4)	1.06989 (18)	0.3008 (2)	0.0345 (6)	
C9	0.5110 (5)	1.10001 (19)	0.3994 (2)	0.0416 (7)	
C10	0.3474 (5)	1.0888 (2)	0.4403 (3)	0.0516 (8)	
H10	0.3383	1.1076	0.5068	0.062*	
C11	0.1954 (5)	1.0497 (2)	0.3829 (3)	0.0536 (8)	
H11	0.0844	1.0422	0.4109	0.064*	
C12	0.2082 (4)	1.0217 (2)	0.2843 (2)	0.0431 (7)	
H12	0.1050	0.9964	0.2455	0.052*	
N1	0.3873 (3)	1.00439 (16)	0.14018 (17)	0.0365 (6)	
H1N	0.467 (4)	1.031 (2)	0.107 (2)	0.044*	
O1	0.4094 (4)	0.89646 (15)	0.00411 (15)	0.0486 (6)	
O2	0.1801 (3)	0.87121 (16)	0.12563 (17)	0.0469 (5)	
Cl1	0.95260 (14)	0.69893 (7)	0.40587 (8)	0.0675 (3)	
Cl2	0.73258 (11)	1.08276 (6)	0.24909 (6)	0.0517 (3)	
Cl3	0.69792 (14)	1.15158 (6)	0.47020 (6)	0.0596 (3)	
S1	0.35957 (10)	0.89965 (5)	0.10606 (5)	0.0353 (2)	

### Atomic displacement parameters (Å^2^)

**Table:**

	*U*^11^	*U*^22^	*U*^33^	*U*^12^	*U*^13^	*U*^23^
C1	0.0408 (15)	0.0311 (13)	0.0289 (13)	−0.0055 (11)	0.0092 (11)	−0.0026 (10)
C2	0.0421 (15)	0.0385 (15)	0.0334 (14)	−0.0047 (12)	0.0137 (12)	−0.0011 (11)
C3	0.0514 (17)	0.0388 (15)	0.0309 (14)	−0.0032 (13)	0.0085 (12)	0.0011 (11)
C4	0.0419 (16)	0.0372 (15)	0.0479 (18)	−0.0019 (12)	−0.0004 (13)	−0.0040 (12)
C5	0.0352 (16)	0.064 (2)	0.067 (2)	−0.0019 (15)	0.0126 (15)	0.0076 (17)
C6	0.0483 (18)	0.059 (2)	0.0478 (18)	−0.0058 (15)	0.0201 (15)	0.0102 (15)
C7	0.0400 (14)	0.0281 (12)	0.0303 (13)	0.0033 (11)	0.0068 (11)	0.0040 (10)
C8	0.0387 (15)	0.0308 (13)	0.0343 (14)	0.0054 (11)	0.0055 (11)	0.0045 (11)
C9	0.0533 (18)	0.0326 (14)	0.0377 (16)	0.0021 (13)	0.0014 (13)	−0.0018 (11)
C10	0.066 (2)	0.0518 (19)	0.0397 (17)	0.0027 (16)	0.0189 (15)	−0.0070 (13)
C11	0.056 (2)	0.056 (2)	0.0528 (19)	−0.0010 (16)	0.0256 (16)	−0.0040 (15)
C12	0.0419 (16)	0.0428 (16)	0.0460 (17)	0.0003 (13)	0.0111 (13)	−0.0012 (13)
N1	0.0441 (13)	0.0362 (13)	0.0302 (12)	−0.0057 (10)	0.0082 (10)	0.0030 (9)
O1	0.0695 (15)	0.0514 (13)	0.0254 (10)	−0.0129 (11)	0.0077 (10)	−0.0030 (8)
O2	0.0413 (12)	0.0525 (13)	0.0460 (12)	−0.0151 (10)	0.0019 (9)	0.0011 (10)
Cl1	0.0594 (6)	0.0690 (6)	0.0686 (6)	0.0082 (4)	−0.0138 (4)	0.0057 (4)
Cl2	0.0380 (4)	0.0687 (6)	0.0490 (5)	−0.0056 (3)	0.0081 (3)	−0.0070 (3)
Cl3	0.0697 (6)	0.0598 (5)	0.0456 (5)	−0.0055 (4)	−0.0068 (4)	−0.0122 (3)
S1	0.0418 (4)	0.0381 (4)	0.0260 (4)	−0.0093 (3)	0.0045 (3)	−0.0011 (2)

### Geometric parameters (Å, °)

**Table:**

C1—C2	1.381 (4)	C7—N1	1.420 (3)
C1—C6	1.386 (4)	C8—C9	1.394 (4)
C1—S1	1.760 (3)	C8—Cl2	1.714 (3)
C2—C3	1.379 (4)	C9—C10	1.369 (5)
C2—H2	0.9300	C9—Cl3	1.724 (3)
C3—C4	1.376 (4)	C10—C11	1.384 (5)
C3—H3	0.9300	C10—H10	0.9300
C4—C5	1.380 (5)	C11—C12	1.379 (4)
C4—Cl1	1.734 (3)	C11—H11	0.9300
C5—C6	1.378 (5)	C12—H12	0.9300
C5—H5	0.9300	N1—S1	1.637 (2)
C6—H6	0.9300	N1—H1N	0.858 (18)
C7—C12	1.384 (4)	O1—S1	1.434 (2)
C7—C8	1.399 (4)	O2—S1	1.418 (2)
			
C2—C1—C6	121.0 (3)	C7—C8—Cl2	119.7 (2)
C2—C1—S1	119.3 (2)	C10—C9—C8	120.4 (3)
C6—C1—S1	119.7 (2)	C10—C9—Cl3	119.9 (2)
C3—C2—C1	119.3 (3)	C8—C9—Cl3	119.7 (2)
C3—C2—H2	120.4	C9—C10—C11	120.1 (3)
C1—C2—H2	120.4	C9—C10—H10	119.9
C4—C3—C2	119.6 (3)	C11—C10—H10	119.9
C4—C3—H3	120.2	C12—C11—C10	120.1 (3)
C2—C3—H3	120.2	C12—C11—H11	120.0
C3—C4—C5	121.4 (3)	C10—C11—H11	120.0
C3—C4—Cl1	119.0 (2)	C11—C12—C7	120.5 (3)
C5—C4—Cl1	119.6 (2)	C11—C12—H12	119.8
C6—C5—C4	119.2 (3)	C7—C12—H12	119.8
C6—C5—H5	120.4	C7—N1—S1	120.50 (18)
C4—C5—H5	120.4	C7—N1—H1N	118 (2)
C5—C6—C1	119.5 (3)	S1—N1—H1N	112 (2)
C5—C6—H6	120.2	O2—S1—O1	120.13 (14)
C1—C6—H6	120.2	O2—S1—N1	108.73 (14)
C12—C7—C8	119.4 (3)	O1—S1—N1	104.63 (12)
C12—C7—N1	120.9 (3)	O2—S1—C1	107.60 (13)
C8—C7—N1	119.6 (2)	O1—S1—C1	108.90 (14)
C9—C8—C7	119.4 (3)	N1—S1—C1	106.03 (13)
C9—C8—Cl2	120.9 (2)		
			
C6—C1—C2—C3	0.9 (4)	C8—C9—C10—C11	−1.8 (5)
S1—C1—C2—C3	−176.7 (2)	Cl3—C9—C10—C11	178.3 (3)
C1—C2—C3—C4	−0.4 (4)	C9—C10—C11—C12	0.0 (5)
C2—C3—C4—C5	−0.5 (5)	C10—C11—C12—C7	1.3 (5)
C2—C3—C4—Cl1	178.6 (2)	C8—C7—C12—C11	−0.8 (4)
C3—C4—C5—C6	0.7 (5)	N1—C7—C12—C11	−178.4 (3)
Cl1—C4—C5—C6	−178.3 (3)	C12—C7—N1—S1	−63.5 (3)
C4—C5—C6—C1	−0.1 (6)	C8—C7—N1—S1	118.9 (2)
C2—C1—C6—C5	−0.7 (5)	C7—N1—S1—O2	58.7 (2)
S1—C1—C6—C5	177.0 (3)	C7—N1—S1—O1	−171.8 (2)
C12—C7—C8—C9	−0.9 (4)	C7—N1—S1—C1	−56.7 (2)
N1—C7—C8—C9	176.7 (2)	C2—C1—S1—O2	−20.9 (3)
C12—C7—C8—Cl2	−179.2 (2)	C6—C1—S1—O2	161.3 (2)
N1—C7—C8—Cl2	−1.6 (3)	C2—C1—S1—O1	−152.6 (2)
C7—C8—C9—C10	2.2 (4)	C6—C1—S1—O1	29.6 (3)
Cl2—C8—C9—C10	−179.5 (2)	C2—C1—S1—N1	95.3 (2)
C7—C8—C9—Cl3	−177.8 (2)	C6—C1—S1—N1	−82.5 (3)
Cl2—C8—C9—Cl3	0.4 (3)		

### Hydrogen-bond geometry (Å, °)

**Table:**

*D*—H···*A*	*D*—H	H···*A*	*D*···*A*	*D*—H···*A*
N1—H1N···O1^i^	0.86 (2)	2.11 (2)	2.944 (3)	163 (3)

Symmetry codes: (i) −*x*+1, −*y*+2, −*z*.
